# [Retracted] (-)-Epicatechin protects against myocardial ischemia-induced cardiac injury via activation of the PTEN/PI3K/AKT pathway

**DOI:** 10.3892/mmr.2026.14036

**Published:** 2026-10-05

**Authors:** Jia-Wen Li, Xiao-Yun Wang, Xin Zhang, Lei Gao, Li-Feng Wang, Xin-Hua Yin

Mol Med Rep 17: 8300–8308, 2018; DOI: 10.3892/mmr.2018.8870

Following the publication of the above paper, it was drawn to the Editor's attention by a concerned reader that the control GAPDH western blot data featured in Fig. 4A on p. 8305 had already appeared previously in an article in the journal *Cellular Physiology and Biochemistry* in a different experimental context written by different authors (albeit one of the research institutes was held in common between the papers). In addition, after assessing the data in this paper independently in the Editorial Office, it also came to light that two pairs of data panels featured respectively in Fig. 2A (showing H&E-stained histological sections) and Fig. 2B (showing the results of TUNEL assays) contained overlapping sections of data where different experimental conditions were reported, suggesting that these data panels were derived from the same original sources.

Owing to the fact that the contentious data in the above article had already been published prior to its submission to *Molecular Medicine Reports*, the Editor has decided that this paper should be retracted from the Journal. Upon contacting the authors, they accepted the decision to retract the paper. The Editor apologizes to the readership for any inconvenience caused.

